# A Case of Cardiac Sarcoidosis Masquerading As Heart Failure With Ventricular Arrhythmia

**DOI:** 10.7759/cureus.36239

**Published:** 2023-03-16

**Authors:** Kain Kim, Charles Marvil, Bhavin B Adhyaru

**Affiliations:** 1 Medicine, Emory University School of Medicine, Atlanta, USA; 2 Internal Medicine, Emory University School of Medicine, Atlanta, USA

**Keywords:** multimodality cardiac imaging, heart failure, cardiac magnetic resonance, ventricular arrhythmia, sarcoidosis

## Abstract

Sarcoidosis is a rare cause of cardiomyopathy that can easily be confused for acute heart failure when pulmonary manifestations are absent. We present the case of a 41-year-old female presenting with dyspnea found to have ventricular arrhythmia on arrival at the emergency department. Cardiac magnetic resonance and computed tomography of the chest with contrast were performed, confirming the systemic sarcoidosis diagnosis with cardiac involvement.

## Introduction

Sarcoidosis is a multisystem inflammatory disease of unknown etiology, characterized by noncaseating granulomas in various organs [1]. More than 90% of patients with sarcoidosis present with lung and intrathoracic lymph node involvement [1]. Notably, the prognosis is worst in cases with cardiac manifestations, accounting for more than two-thirds of global deaths due to sarcoidosis [2]. Diagnosis of sarcoidosis can be challenging due to highly variable clinical presentation and non-specific symptoms, leading to many cases being diagnosed through incidental findings. The relative rarity of cardiac sarcoidosis (CS), occurring in approximately 5% of patients with systemic sarcoidosis, makes screening especially imperative [3]. Prior guidelines focused primarily on using endomyocardial biopsy for diagnosis, but this is a high-risk procedure only sometimes offered at resource-limited institutions. In 2016, updated guidelines focusing on using advanced imaging modalities instead were released by the Japanese Circulation Society Guideline on Diagnosis and Treatment of Cardiac Sarcoidosis [4].

## Case presentation

**Figure 1:**
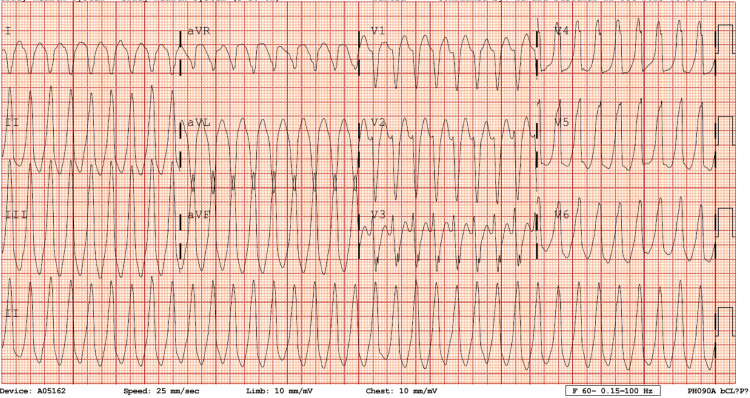
Initial ECG Initial Electrocardiogram showing regular, monomorphic wide-complex tachycardia. Unable to determine axis due to lead limb reversal in aVR and aVL. No clear AV dissociations. Unable to distinguish supraventricular tachycardia from aberrancy and ventricular tachycardia. aVR: augmented Vector Right; aVL augmented Vector Left; AV: atrioventricular

A 41-year-old female with a past medical history of hypertension presented with sudden palpitations, shortness of breath, and diaphoresis shortly after attending a car racing event where she was consuming alcohol. On admission to the emergency department, she was found to have a heart rate of 219 bpm with stable blood pressure and slight dyspnea. Otherwise, she was hemodynamically stable with an unremarkable physical exam. Her labs showed a 0.06 ng/mL troponin, Brain natriuretic peptide (BNP) of 432 pg/mL, and normal electrolytes. Her initial electrocardiogram (ECG) showed a wide-complex tachycardia with a rate of 250 bpm (Figure 1).

**Figure 2:**
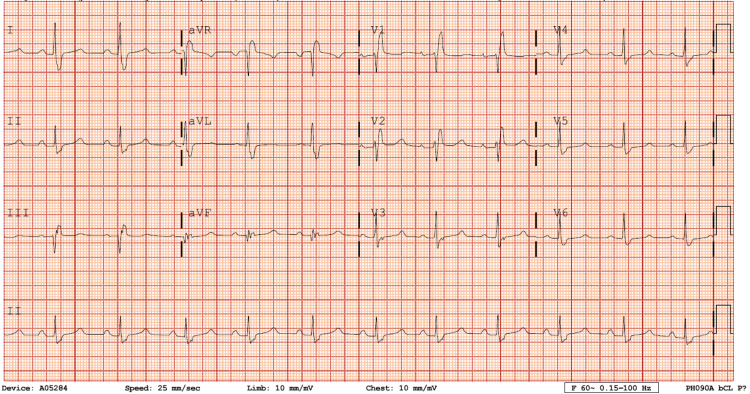
Electrocardiogram after cardioversion showing right bundle branch block pattern and left atrial enlargement.

**Figure 3:**
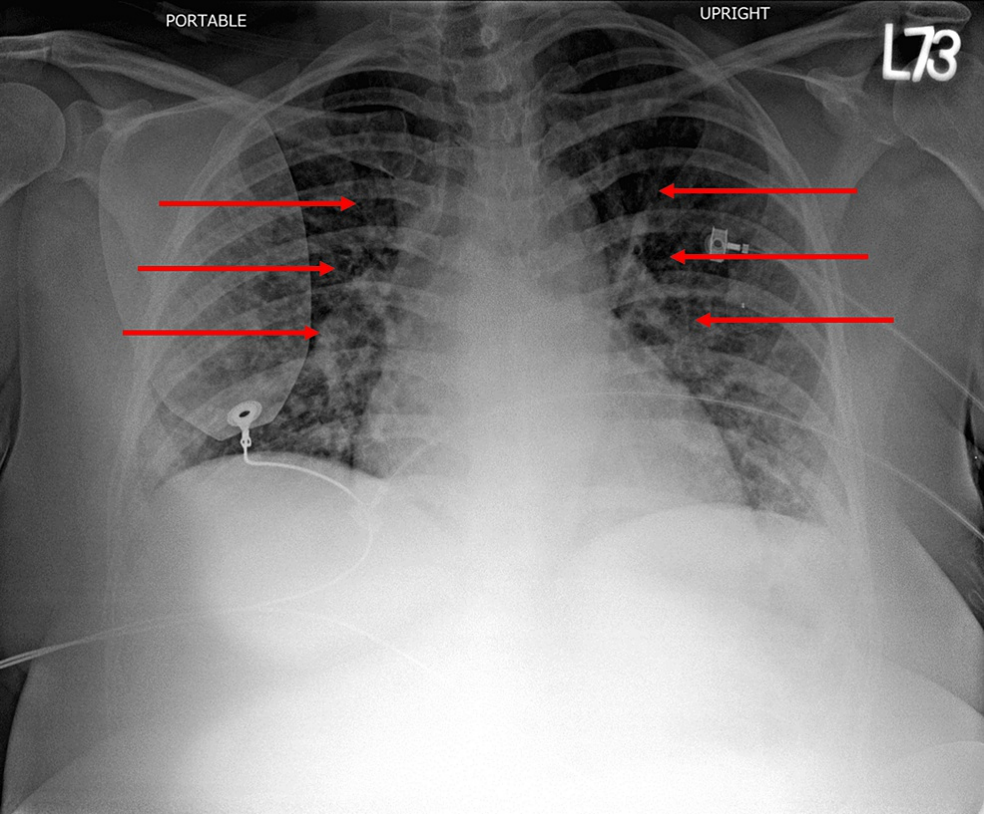
Chest X-ray showing enlarged cardiac silhouette with interstitial infiltrates.

Her tachycardia did not break after receiving carotid massage, vagal maneuver, or chemical cardioversion with amiodarone but did break with electrical cardioversion. It was difficult to discern whether the patient had ventricular tachycardia versus supraventricular tachycardia (SVT) with aberrancy. The underlying ECG rhythm showed a right bundle branch block (RBBB) pattern and some left atrial (LA) enlargement (Figure 2). Her chest X-ray showed possible pulmonary edema (Figure 3). She was admitted to the hospital for closer monitoring and work-up.

On further questioning, the patient reported recently having three pillow orthopnea and dyspnea on exertion. A transthoracic echocardiogram showed an ejection fraction of 30-35%, a mildly dilated left ventricular (LV) cavity, hypokinesis of the basal anterior and anteroseptal segments the LV, stage 2 diastolic dysfunction, a mildly dilated LA, and a normal RV. Given her initial presentation and concern for heart failure, the medical team decided to pursue left heart catheterization for possible obstructive coronary artery disease (CAD), the results of which revealed no vascular disease. 

**Figure 4:**
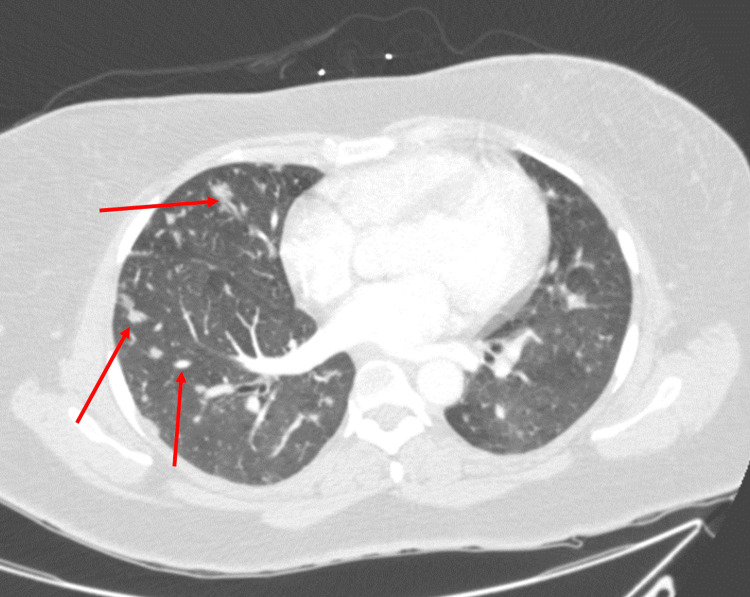
Computed tomography angiography of the chest with contrast demonstrating innumerable sub-centimeter nodules in a perilymphatic distribution.

**Figure 5:**
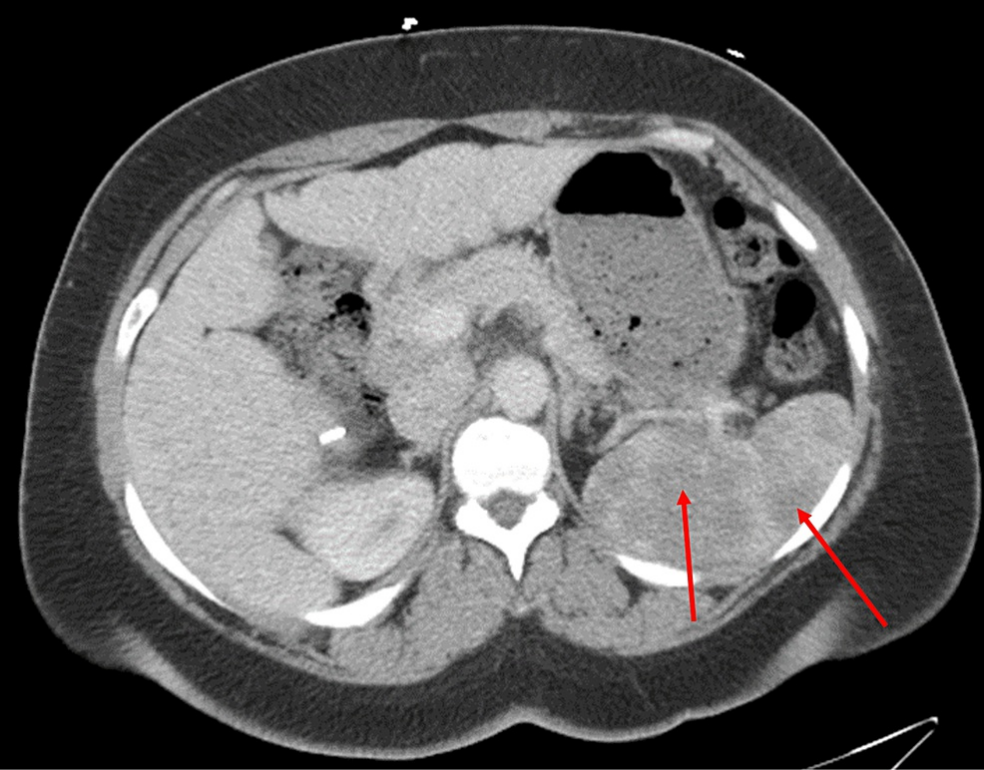
Computed tomography angiography of the abdomen/pelvis showing multiple non-specific hypoattenuating lesions throughout the spleen and heterogeneous liver with nodular morphology.

Given an unclear diagnosis with findings of heart failure, a computed tomography (CT) of the chest with contrast was completed. It showed innumerable sub-centimeter nodules in a perilymphatic distribution (Figure 4). Given the new possibility of sarcoidosis or malignancy, a CT of the abdomen/pelvis was completed, revealing multiple non-specific hypoattenuating lesions throughout the spleen and heterogeneous liver with nodular morphology (Figure 5).

**Figure 6:**
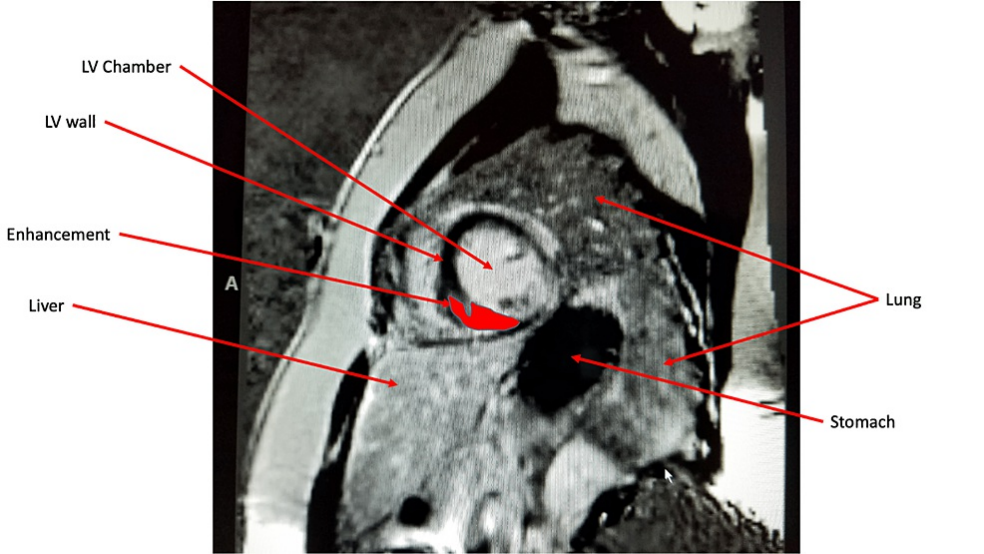
Cardiac MRI. Late gadolinium enhancement in the left ventricular wall.

**Figure 7:**
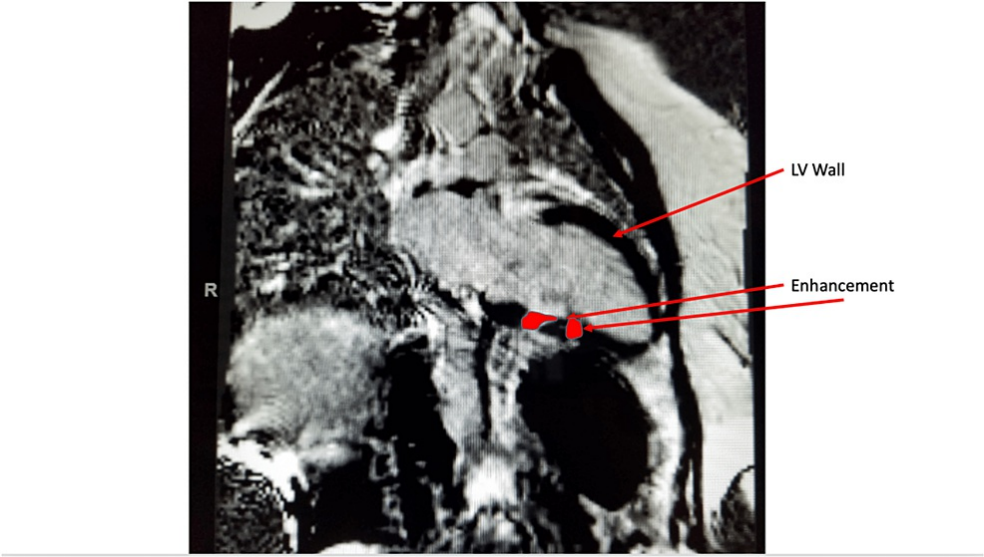
Cardiac MRI. Late gadolinium enhancement in the left ventricular wall.

**Figure 8:**
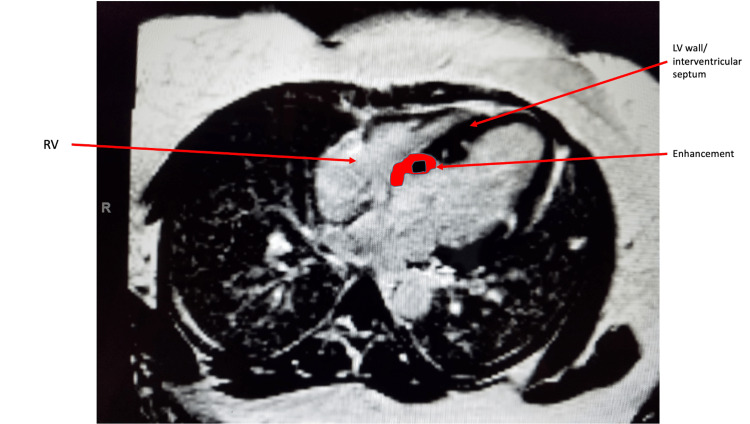
Cardiac MRI. Late gadolinium enhancement in the left ventricular wall.

Given the constellation of findings, there was a concern for systemic sarcoidosis with cardiac involvement. A fludeoxyglucose (FDG) positron emission tomography (PET) was unavailable in the inpatient setting. As per cardiology consult recommendations, the patient subsequently had magnetic resonance imaging (MRI) of cardiac morphology and function with delayed gadolinium, which showed patchy abnormal mid-myocardial delayed enhancement in the left ventricular myocardium (Figure 6-8). This was compatible with changes in myocardial sarcoid and confirmed the diagnosis of CS. She received implantation of an Automatic Implantable Cardioverter Defibrillator (AICD) because of her arrhythmia and heart failure and was discharged on high-dose steroids.

## Discussion

We present a case of what initially appeared to be an arrhythmia related to new-onset heart failure, presenting with cardiac symptoms without outward evidence of sarcoidosis involvement in other organ systems (no rash, uveitis, hepatosplenomegaly, etc.). On further investigation, however, imaging revealed perilymphatic nodules and lesions throughout the spleen and liver consistent with sarcoidosis. True isolated CS is rare (prevalence < 10%), and its existence itself is controversial, as sarcoidosis is, by definition, a systemic disease [5]. Recent findings suggest that isolated CS may present with more severe cardiac manifestations than non-isolated CS, although there are no detectable differences in long-term survival with treatment [6]. 

Though 20-25% of patients with pulmonary or systemic sarcoidosis are estimated to have some cardiac involvement, only about 5% will develop clinically manifest cardiac features [3,7]. Indeed, in a recent investigation of 351 CS patients, 54 out of 84 recorded deaths (64%) were due to unexpected sudden cardiac death, in which CS has only been diagnosed postmortem [8]. The largely asymptomatic nature of cardiac sarcoidosis is especially concerning, considering that its incidence has also increased in the last few years, likely due in part to technological advances in cardiac imaging. However, the potential contributions of the changing epidemiology of the disease are yet unknown [9-10]. Finally, there is an average delay of nearly two years from symptom onset to diagnosis, further necessitating an elevated level of clinical suspicion and proactive investigation [11].

Common presenting features of clinically manifest CS include conduction abnormalities like AV block, ventricular arrhythmia (VA), and heart failure, consistent with our patient's presentation [12]. Even in cases such as ours that mimic traditional arrhythmogenic right ventricular cardiomyopathy (ARVC), it is crucial to exclude CS as a possible diagnosis; as demonstrated in a prospective comparison study, Dechering et al. showed that 5 of 8 patients with established CS also fulfilled criteria for a diagnosis of ARVC [13]. 

CS should be suspected when dyspnea appears disproportionate to the degree of apparent lung involvement. An initial baseline ECG is recommended in all sarcoidosis patients and may reveal ventricular atopy, left or right bundle branch block, pathologic Q waves, or ventricular arrhythmia. The recommended work-up includes a transthoracic echocardiogram and cardiac MRI or PET CT if MRI is unavailable/ineffective or when renal dysfunction precludes Gadolinium contrast agents [14]. MRI and PET imaging are recommended due to PET's ability to identify areas of pathologic glucose uptake and inflammation, which indicates disease activity and the need for treatment with high-dose steroids [15]. ECG findings may include left ventricular or diastolic dysfunction, while MRI findings include LV systolic dysfunction. The presence of delayed Gadolinium enhancement with abnormal patchy pattern has high sensitivity (92-100%) and specificity (78-100%) for CS [16-18].

Though difficult, diagnosis of CS can be accomplished through one of two ways: 1. a histological diagnosis from myocardial tissue, or 2. clinical diagnosis after negative myocardial biopsy (or exclusion of feasible myocardial biopsy). The latter requires at least one or more of the following: steroid +/- immunosuppressant responsive cardiomyopathy or heart block, unexplained reduced LV ejection fraction (<40%), unexplained sustained (spontaneous or induced) ventricular tachycardia, Mobitz type II second-degree heart block or third-degree heart block, patchy uptake on dedicated cardiac PET, late gadolinium enhancement on cardiac MRI, positive gallium uptake, and reasonable exclusion of other causes for cardiac manifestations.

The first-line treatment for CS is corticosteroid therapy, which may improve survival but has not shown a reduction in the incidence of VA [19]. ICD placement is recommended in patients with sarcoidosis and a history of nonsustained ventricular tachycardia, and a cardiac transplant is also an option for younger patients [20].

## Conclusions

The diagnosis of CS is challenging, mainly when presenting with symptoms of heart failure without pulmonary manifestations. As demonstrated in our case, patients with CS are at elevated risk of ventricular arrhythmias, which are also more difficult to predict. In presenting this case study, we hope to encourage early clinical suspicion for CS in cases of arrhythmia by highlighting the high incidence of VA in this clinically variable condition.
